# Serum Folate, Vitamin B12 Levels, and Systemic Immune-Inflammation Index Correlate With Motor Performance in Parkinson's Disease: A Cross-Sectional Study

**DOI:** 10.3389/fneur.2021.665075

**Published:** 2021-05-21

**Authors:** Siying Li, Qingxi Zhang, Yuyuan Gao, Kun Nie, Yanling Liang, Yuhu Zhang, Lijuan Wang

**Affiliations:** ^1^Department of Neurology, Guangdong Neuroscience Institute, Guangdong Provincial People's Hospital, Guangdong Academy of Medical Sciences, Guangzhou, China; ^2^Department of Neurology, The Third Affiliated Hospital of Guangzhou Medical University, Guangzhou, China

**Keywords:** Parkinson's disease, folate, VitB12, inflammation, cross-sectional study

## Abstract

This study aimed to investigate the influence of serum folate, vitamin B12 (VitB12) levels, and inflammation-based scores on the motor performance status in Parkinson's disease (PD). We retrospectively collected data from 148 consecutive patients with idiopathic PD first admitted to our hospital. We measured whole blood count, albumin, lactate dehydrogenase, C-reactive protein, folate, and VitB12 levels and calculated the inflammation-based scores. The following scales were applied to assess the motor performance status: activity of daily living scale (ADL, the Barthel Index), the Unified Parkinson's Disease Rating Scale Part III (UPDRS-III), and Hoehn–Yahr (H–Y) classification. The geometric mean of folate and VitB12 levels were 11.87 (ng/ml) and 330.52 (pmol/L), respectively. Folate deficiency (serum level < 4.0 ng/ml) and VitB12 deficiency (serum level < 133 pg/ml) were present in 0.7 and 5.4% of the patients, respectively. The mean prognostic nutritional index (PNI) and systemic immune-inflammation index (SII) were 47.78 ± 4.42 and 470.81 ± 254.05, respectively. The multivariate analyses showed that serum VitB12 level (*P* = 0.002) and SII (*P* = 0.005) were significant factors for ADL score; serum folate (*P* = 0.027) and VitB12 (*P* = 0.037) levels for UPDRS-III score; and serum folate (*P* = 0.066) and VitB12 (*P* = 0.017) levels for H–Y classification. The elevated folate level did correlate with greater decline in UPDRS-III score (*P* = 0.023) and H–Y classification (*P* = 0.003), whereas there was an obvious increase in ADL score (*P* = 0.048). SII was negatively associated (*P* < 0.001) with the ADL score. The three-dimensional drawing, combined with the effect of folate and VitB12 levels, showed that the lowest level of folate was associated with the lowest ADL score and the highest UPDRS-III score and H–Y classification. This study indicates that serum folate, VitB12 levels, and SII are significant factors influencing the motor performance status in patients with PD. SII is negatively associated with ADL. Elevated serum folate level correlates with mild motor impairment in patients with PD.

## Introduction

Parkinson's disease (PD) is the second most common neurodegenerative disorder, with ~700,000 predicted cases in the United States from 2000 to 2040 (1). The main pathological features of PD are the loss of dopamine (DA)-ergic neurons in the substantia nigra (SN) and the presence of Lewy bodies (LBs), which are composed mainly of alpha-synuclein (2). Previous studies have demonstrated that protein misfolding and aggregation, disruption of autophagic catabolism, mitochondrial dysfunction, and loss of calcium homeostasis are the critical factors leading to the death of SN DAergic neurons (3).

The cell-autonomous mechanisms of neurodegeneration, which arise from an interaction between genetic and environmental risk factors, play an important role in the development of PD (4, 5). However, the pathological progression of PD involves several non-cell-autonomous mechanisms that facilitate further degeneration. Principal among these non-cell-autonomous factors is neuroinflammation, which involves a series of immune-mediated cascades that are triggered by the loss of DAergic neurons (4, 5). *In vivo* experiments have revealed that glial cells, particularly microglia, can adopt a pro-inflammatory phenotype that is associated with the release of cytotoxic molecules, which lead to enhanced neurodegeneration (4). Our previous study indicated that macrophage migration inhibitory factor (MIF) mediated neuroprotective effects *via* suppressing inflammatory responses in PD. These findings might demonstrate a causal link between neuroinflammation and PD pathogenesis (6).

The blood–brain barrier was once thought to inhibit the entry of inflammatory cells and mediators from the peripheral circulation, but increasing evidence suggests that the blood–brain barrier is affected due to pathological conditions (7, 8). Therefore, the status of systemic inflammation can partially reflect the progress and prognosis of PD. Many inflammation-based prognostic scores were developed on the basis of systemic inflammatory responses, such as the Glasgow Prognostic Score (GPS) and modified Glasgow Prognostic Score (mGPS), both evaluated by combining serum C-reactive protein (CRP) and albumin (ALB) levels; Prognostic Nutritional Index (PNI), measured by the ALB level; and the lymphocyte count measured by neutrophil-to-lymphocyte ratio (NLR), platelet-to-lymphocyte ratio (PLR), and lymphocyte-to-monocyte ratio (LMR); and the SII, calculated by the neutrophil, lymphocyte, and platelet counts. These scores have shown the prognostic value in several types of cancer (9–11) and immune-inflammation-related diseases (12).

Folate is particularly critical during the early development of the brain and is essential for the maintenance of normal brain function during the later stages of development. Folate and vitamin B12 (VitB12) are required for the methionine synthase reaction in which a methyl group is transferred from methyltetrahydrofolate (methyl-H4-folate; also known as levomefolic acid) to homocysteine by methionine synthase, with methyl-VitB12 as a coenzyme to form methionine. The resulting H4-folate can then be returned to the folate pool and can be made available for the generation of methylene-H4-folate, the form required for *de novo* synthesis of thymidine, which is essential for DNA replication and repair. Hence, either folate or B12 deficiency results in the same biochemical perturbation in thymidine synthesis and DNA replication (13). Folate and VitB12 deficiency results in an increase in blood and intracellular levels of homocysteine. Homocysteine is readily and partly reversibly transformed into other compounds, termed as “total homocysteine (tHcy).” Folate deficiency and tHcy impair DNA repair in hippocampal neurons and sensitize them to oxidative damage (14). Low serum VitB12 level was associated with an accelerated rate of white matter damage and brain atrophy (15–17). A prospective study that included 5,289 participants with a mean follow-up of 9.7 years failed to identify any relationship between VitB12 level and the risk of development of PD (18). Conversely, a large cohort collected from participants with a double-blind, randomized trial of PD (DATATOP) showed that low VitB12 levels at baseline predicted greater worsening of mobility, meanwhile elevated tHcy predicted greater cognitive decline (19).

There might be an indirect regulation between folate/VitB12 and the inflammatory cells and mediators. Previous studies suggested that cobalamin (VitB12) conversely decreased the expression of neurotrophic epidermal growth factor (EGF) and increased the expression of neurotoxic tumor necrosis factor-alpha (TNF-alpha) both in serum and cerebrospinal fluid (CSF) (20, 21). The increase in TNF-alpha and NGF levels increased the nuclear factor-kappa B activity levels and indirectly modulated the expression of some neuroinflammatory cytokines (13).

This study aimed to investigate the influence of serum folate, VitB12 levels, and inflammation-based scores on the motor performance status in patients with PD.

## Materials and Methods

### Ethical Statement

This study was performed with the approval of the Ethics Committee of the Third Affiliated Hospital of Guangzhou Medical University and the Ethics Committee of Guangdong Provincial People's Hospital (No. 2021-006). Informed consent was obtained from all patients. This study has been conducted according to the principles expressed in the Declaration of Helsinki. All the collected data were stored according to the ethical guidelines of medical research.

### Study Participants

We retrospectively collected data from 148 consecutive patients with idiopathic PD admitted to our hospital for the first time from January 2018 to June 2020 so that data could be collected before dopaminergic medication. Diagnosis of PD was evaluated by two experienced neurologists, strictly according to the United Kingdom Parkinson's Disease Society Brain Bank Clinical Diagnostic Criteria. Exclusion criteria were as follows: PD induced by chemical drugs, trauma, cerebrovascular disease, inflammatory bowel disease, and other reasons; patients with PD complicated with nephropathy, tumor, myocardial infarction, stroke, and recent infection or surgery; patients with PD complicated with non-neurodegenerative (Alzheimer's disease or cognitive impairments) nervous system disease; and patients with missing outcome assessment medical record were excluded. We collected data on patient characteristics, such as education status, smoking, alcohol consumption, diabetes mellitus, ABO blood type, PD duration, whole blood count, ALB, total bilirubin (TBIL), alanine transaminase (ALT), creatinine (Cr), uric acid (UA), lactate dehydrogenase (LDH), CRP, prothrombin time (PT), D-dimmer (D-D), fibrinogen, folate, and VitB12 levels. Serum folate level < 4.0 ng/mL and VitB12 level < 133.0 pg/mL were considered as deficient. The outcome assessment or PD performance status was assessed by the activity of daily living scale (ADL), the Unified Parkinson's Disease Rating Scale Part III (UPDRS-III), and the Hoehn–Yahr (H–Y) classification. We used the Barthel Index (BI) to measure the basic ADL function (self-maintenance skills, such as dressing, bathing, and grooming) of patients with PD. The BI consists of 10 items and is predominantly used with the patient population (22). The UPDRS-III is the most frequently used scale in the clinical motor examination of PD. Part of the UPDRS-III scores was extracted from the levodopa challenge test before taking Madopar. The test is widely used to assess motor symptom alleviation in unifying the drug administration (23). H–Y classification was commonly used to assess the severity and the stage of motor performance in patients with PD.

### Inflammation-Based Prognostic Scores

Blood samples with routine laboratory measurements of CRP and ALB levels and white blood cell, neutrophil, lymphocyte, monocyte, and platelet counts were obtained at the time of recurrence. The GPS, mGPS, PNI, NLR, PLR, LMR, and SII scores were calculated and constructed as described in Table 1.

**Table 1:** Definition of the systemic inflammation-based scores.

**Scoring systems**	**Score**
Glasgow Prognostic Score (GPS)	
CRP (≤10 mg/L) and ALB (≥35 g/L)	0
CRP (≤10 mg/L) and ALB (<35 g/L)	1
CRP (>10 mg/L) and ALB (≥35 g/L)	1
CRP (>10 mg/L) and ALB (<35 g/L)	2
Modified Glasgow Prognostic Score (mGPS)	
CRP (≤10 mg/L) and ALB (≥35 g/L)	0
CRP (≤10 mg/L) and ALB (<35 g/L)	0
CRP (>10 mg/L)	1
CRP (>10 mg/L) and ALB (<35 g/L)	2
Prognostic Nutritional Index (PNI)	ALB (g/L) + 5 × lymphocyte count (×10^9^/L)
Neutrophil to lymphocyte ratio (NLR)	Neutrophil count (×10^9^/L): lymphocyte count (×10^9^/L)
Platelet to lymphocyte ratio (PLR)	Platelet count (×10^9^/L): lymphocyte count (×10^9^/L)
Lymphocyte to monocyte ratio (LMR)	Lymphocyte count (×10^9^/L): monocyte count (×10^9^/L)
Systemic Immune-inflammation Index (SII)	Platelet count (×10^9^/L) × neutrophil count (×10^9^/L)/lymphocyte count (×10^9^/L)

*CRP, C-reactive protein; ALB, albumin*.

### Statistical Analysis

Continuous data were recorded as mean ± SD. Incidence of nominal variables was reported as the number of cases (percentage). Analysis of variance (ANOVA) and the Chi-square test were used to compare continuous and categorical variables normally distributed, respectively, and non-parametric tests were used for non-normally distributed variables. Variables that achieved statistical significance in the univariate analysis were introduced into the multivariate regression analyses, linear regression analyses for ADL score and UPDRS-III score, and logistic regression analyses for H–Y classification. The crude correlation between independent variables and outcomes was reported in scatter-plot figures. Independent variables were also classified in tertiles to study their combined association with outcomes. SPSS v.23.0 statistical software (SPSS, Inc., Chicago, IL, United States) was used for statistical analysis. *P* < 0.05 was considered as a statistically significant difference.

## Results

### Patient Characteristics

Table 2A shows the demographics and clinical characteristics (including the inflammation-based scores) of the enrolled patients. A total of 148 patients were included in this study. The total population consisted of 56 (37.84%) males and 92 (62.16%) females with a mean age of 63.79 years. The mean interval from the onset of PD-related symptoms to seeking medical care in our hospital was 58.89 months. The geometric mean of WBC, ALB, LDH, CRP, folate, and VitB12 levels were 6.08 (10^9^/L), 39.45 (g/L), 196.18 (U/L), 2.73 (mg/L), 11.87 (ng/mL), and 330.52 (pmol/L), respectively. Folate deficiency (serum level < 4.0 ng/mL) and VitB12 deficiency (serum level < 133 pg/mL) were present in 0.7% and 5.4% of the patients, respectively. Thus, further analysis of folate and VitB12 levels were stratified to tertiles. The mean PNI, NLR, PLR, LMR, and SII were 47.78 ± 4.42, 2.12 ± 0.87, 128.48 ± 52.10, 4.16 ± 1.30, and 470.81 ± 254.05, respectively. Patients were divided into three groups according to the H–Y classification: early stage (1–2 classification), medium stage (2.5–3 classification), and advanced stage (4–5 classification). Table 2B showed that H–Y classification was highly correlated with PD duration, ADL scores, and UPDRS-III scores.

**Table 2A:** Demographic and clinical characteristics of the enrolled patients (*n* = 148).

**Variables**	**Values (*n =* 148)**
Gender (Female/Male)	56/92 (37.84/62.16)
Age (years), mean ± SD	63.79 ± 11.07
Education status (0/1/2/3/4/5)	6/26/44/31/39/2 (4.05/17.57/29.73/20.95/26.35/1.35)
Smoking (Yes/No)	10/138 (6.76/93.24)
Alcohol consumption (Yes/No)	3/145 (2.03/97.97)
Diabetes mellitus (Yes/No)	16/132 (10.81/89.19)
ABO blood type (A/B/AB/O)	34/40/18/56 (22.97/27.03/12.16/37.84)
PD duration (months), mean ± SD	58.89 ± 51.23
WBC (10^9^/L), mean ± SD	6.08 ± 1.55
HGB (g/L), mean ± SD	132.99 ± 15.64
Neutrophil (10^9^/L), mean ± SD	3.62 ± 1.25
Lymphocyte (10^9^/L), mean ± SD	1.80 ± 0.49
Monocyte (10^9^/L), mean ± SD	0.47 ± 0.17
PLT (10^9^/L), mean ± SD	218.51 ± 63.00
ALB (g/L), mean ± SD	39.45 ± 3.59
TBIL (μmol/L), mean ± SD	13.68 ± 5.68
ALT (U/L), mean ± SD	17.17 ± 10.70
Cr (μmol/L), mean ± SD	71.25 ± 16.60
UA (μmol/L), mean ± SD	324.01 ± 95.50
LDH (U/L), mean ± SD	196.18 ± 129.33
CRP (mg/L), mean ± SD	2.73 ± 4.74
PT (s), mean ± SD	13.59 ± 0.94
D-dimmer (ng/ml), mean ± SD	559.53 ± 589.94
Fibrinogen (g/L), mean ± SD	3.28 ± 1.27
Folate (ng/ml), mean ± SD	11.87 ± 5.36
VitB12 (pmol/L), mean ± SD	330.52 ± 197.10
UA/Cr, mean ± SD	4.72 ± 1.50
GPS (0/1/2)	131/15/2 (88.51/10.14//1.35)
mGPS (0/1/2)	136/10/2 (91.89/6.76/1.35)
PNI, mean ± SD	47.78 ± 4.42
NLR, mean ± SD	2.12 ± 0.87
PLR, mean ± SD	128.48 ± 52.10
LMR, mean ± SD	4.16 ± 1.30
SII, mean ± SD	470.81 ± 254.05

*Values are presented as the mean ± SD or n (%)*.

*PD, Parkinson's disease; WBC, white blood cell; HGB, hemoglobin; PLT, platelet; ALB, albumin; TBIL, total bilirubin; ALT, alanine transaminase; Cr, creatinine; UA, uric acid; LDH, lactate dehydrogenase; CRP, C-reactive protein; PT, prothrombin time; VitB12, vitamin B12; GPS, Glasgow Prognostic Score; mGPS, modified Glasgow Prognostic Score; PNI, prognostic nutritional index; NLR, neutrophil-to-lymphocyte ratio; PLr, platelet-to-lymphocyte ratio; LMR, lymphocyte-to-monocyte ratio; SII, systemic immune-inflammation index*.

**Table 2B:** Disease duration, ADL scores, and UPDRS-III scores according to H-Y classification in PD patients (*n* = 148).

**H-Y classification**	**Early stage**	**Medium stage**	**Advanced stage**	***Pearson correlation P***
Cases	78	50	20	
PD duration (months)	34.55 ± 32.82	76.02 ± 45.36	110.95 ± 67.64	<0.001
ADL scores	97.92 ± 4.28	82.50 ± 19.93	61.75 ± 21.72	<0.001
UPDRS-III scores	29.88 ± 11.88	51.40 ± 8.68	68.75 ± 4.24	<0.001

*PD, Parkinson's disease; ADL, activity of daily living scale; UPDRS-III, the Unified Parkinson's Disease Rating Scale Part III; H-Y classification, Hoehn–Yahr classification*.

### Univariate and Multivariate Regression Analyses

The univariate analysis identified that age, diabetes mellitus, PD duration, HGB, lymphocyte, ALB, ALT, CRP, D-D, FIB, folate, VitB12 levels, PNI, NLR, PLR, LMR, and SII were significant factors associated with the ADL score. After the multivariate linear regression analyses, we found that only diabetes mellitus (HR = −11.995; 95% CI = −20.260–3.731; *P* = 0.005), PD duration (HR = −0.113; 95% CI = −0.166–0.060; *P* < 0.001), VitB12 level (HR = −0.021; 95% CI = −0.034–0.008; *P* = 0.002), NLR (HR = −9.370; 95% CI = −0.226–18.513; *P* = 0.045), and SII (HR = −0.059; 95% CI = −0.100–0.018; *P* = 0.005) were significant factors for ADL scores (Table 3).

**Table 3:** Univariate and multivariate regression analyses of the clinical characteristics for ADL scores in PD patients (*n* = 148).

**Univariate**	**HR (95% CI)**	***P***	**Multivariate**	**HR (95% CI)**	***P***
Gender	3.455 (−2.890–9.800)	0.284			
Age	−0.579 (−0.842–0.315)	**<0.001**	Age	−0.226 (−0.490–0.037)	0.090
Education: illiteracy	Reference				
Education: primary	7.288 (−9.682–24.258)	0.397			
Education: junior	6.402 (−9.904–22.708)	0.439			
Education: senior	−2.070 (−18.781–14.641)	0.807			
Education: undergraduate	3.397 (−13.034–19.828)	0.683			
Education status: post-graduate	0.833 (−29.759–31.426)	0.957			
Smoking	5.552 (−6.723–17.827)	0.373			
Alcohol consumption	12.429 (−9.400–34.258)	0.262			
Diabetes mellitus	−12.275 (−22.020–2.530)	**0.014**	Diabetes mellitus	−11.995 (−20.260–3.731)	**0.005**
PD duration	−0.159 (−0.213–0.104)	**<0.001**	PD duration	−0.113 (−0.166–0.060)	**<0.001**
ABO blood type: A	Reference				
ABO blood type: B	−2.392 (−11.160–6.376)	0.591			
ABO blood type: AB	3.969 (−6.988–14.926)	0.475			
ABO blood type: O	2.233 (−5.939–10.406)	0.590			
WBC	−0.903 (−2.902–1.096)	0.373			
HGB	0.220 (0.025–0.415)	**0.027**	HGB	−0.045 (−0.231–0.142)	0.636
Lymphocyte	5.723 (−0.543–11.989)	**0.073**	Lymphocyte	12.234 (−4.047–23.758)	0.163
Neutrophil	−1.935 (−4.392–0.522)	0.122			
Monocyte	−12.662 (−30.706–5.382)	0.168			
Platelet	−0.034 (−0.082–0.015)	0.177			
ALB	1.373 (0.538–2.208)	**0.001**	ALB	0.476 (−0.342–1.293)	0.252
TBIL	0.303 (−0.240–0.847)	0.272			
ALT	0.341 (0.057–0.625)	**0.019**	ALT	0.022 (−0.224–0.267)	0.862
CREA	0.023 (−0.164–0.210)	0.810			
URIC	0.007 (−0.025–0.040)	0.661			
LDH	−0.014 (−0.038–0.010)	0.250			
CRP	−1.090 (−1.719–0.460)	**0.001**	CRP	−0.102 (−0.786–0.583)	0.770
PT	1.430 (−1.857–4.717)	0.391			
D-D	−0.010 (−0.015–0.005)	**<0.001**	D-D	−0.004 (−0.009–0.000)	0.071
FIB	−2.054 (−4.468–0.359)	**0.095**	FIB	−1.482 (−3.590–0.626)	0.167
Folate	0.592 (0.022–1.162)	**0.042**	Folate	0.394 (−0.086–0.873)	0.107
VitB12	−0.026 (−0.041–0.011)	**0.001**	VitB12	−0.021 (−0.034–0.008)	**0.002**
GPS = 0.0	Reference				
GPS = 1.0	−4.882 (−12.095–2.331)	0.183			
GPS = 2.0	−32.912 (−54.217–11.608)	**0.003**	GPS = 2.0	2.865 (−27.793–33.522)	0.854
mGPS = 0.0	Reference				
mGPS = 1.0	−12.850 (−29.264–3.564)	0.124			
mGPS = 2.0	−43.850 (−69.534–18.165)	**0.001**	mGPS = 2.0	−24.048 (−65.454–17.358)	0.253
PNI	1.291 (0.612–1.971)	**<0.001**	PNI	0.476 (−0.342–1.293)	0.252
NLR	−4.264 (−7.563–0.965)	**0.012**	NLR	9.370 (0.226–18.513)	**0.045**
PLR	−0.092 (−0.157–0.027)	**0.006**	PLR	0.128 (−0.030–0.286)	0.112
LMR	1.812 (−0.241–3.864)	**0.083**	LMR	−0.540 (−2.593–1.514)	0.604
SII	−0.020 (−0.032–0.008)	**0.001**	SII	−0.059 (−0.100–0.018)	**0.005**
UA/Cr	0.697 (−1.402–2.797)	0.512			

*ADL, activity of daily living scale; PD, Parkinson's disease; HR, hazard ratio; CI, confidence interval; WBC, white blood cell; HGB, hemoglobin; PLT, platelet; ALB, albumin; TBIL, total bilirubin; ALT, alanine transaminase; Cr, creatinine; UA, uric acid; LDH, lactate dehydrogenase; CRP, C-reactive protein; PT, prothrombin time; VitB12, vitamin B12; GPS, Glasgow Prognostic Score; mGPS, modified Glasgow Prognostic Score; PNI, prognostic nutritional index; NLR, neutrophil-to-lymphocyte ratio; PLR, platelet-to-lymphocyte ratio; LMR, lymphocyte-to-monocyte rati; SII, systemic immune-inflammation index*.

The univariate analysis identified that age, PD duration, B blood type, lymphocyte, TBIL, CRP, D-D, folate, VitB12 levels, PNI, and NLR were significant factors associated with UPDRS-III score. After the multivariate linear regression analyses, we found that only age (HR = 0.348; 95% CI = 0.096–0.601; *P* = 0.007), PD duration (HR = 0.124; 95% CI = 0.075–0.174; *P* < 0.001), folate level (HR = −0.519; 95% CI = −0.979–0.060; *P* = 0.027), and VitB12 level (HR = 0.013; 95% CI = 0.01–0.026; *P* = 0.037) were significant factors for UPDRS-III scores (Table 4).

**Table 4:** Univariate and multivariate regression analyses of the clinical characteristics for UPDRS-III scores in PD patients (*n* = 148).

**Univariate**	**HR (95% CI)**	***P***	**Multivariate**	**HR (95% CI)**	***P***
Gender	0.422 (−5.461–6.306)	0.887			
Age	0.478 (0.231–0.724)	**<0.001**	Age	0.348 (0.096–0.601)	**0.007**
Education: illiteracy	Reference				
Education: primary	−2.885 (−18.693–12.924)	0.719			
Education: junior	−6.318 (−21.509–8.872)	0.412			
Education: senior	−4.177 (−19.745–11.391)	0.597			
Education: undergraduate	−7.244 (−22.550–8.063)	0.351			
Education status: post-graduate	5.500 (−23.000–34.000)	0.703			
Smoking	−6.333 (−17.655–4.989)	0.271			
Alcohol consumption	−8.920 (−29.117–11.278)	0.384			
Diabetes mellitus	5.362 (−3.787–14.510)	0.249			
PD duration	0.161 (0.112–0.210)	**<0.001**	PD duration	0.124 (0.075–0.174)	**<0.001**
ABO blood type: A	Reference				
ABO blood type: B	6.972 (−1.098–15.042)	**0.090**	ABO blood type: B	2.218 (−3.188–7.625)	0.419
ABO blood type: AB	2.147 (−7.938–12.232)	0.675			
ABO blood type: O	3.718 (−3.803–11.240)	0.330			
WBC	0.231 (−1.620–2.082)	0.805			
HGB	−0.093 (−0.276–0.089)	0.313			
Lymphocyte	−5.247 (−11.036–0.542)	**0.075**	Lymphocyte	−4.215 (−12.086–3.656)	0.291
Neutrophil	1.016 (−1.266–3.299)	0.380			
Monocyte	6.717 (−10.023–23.457)	0.429			
Platelet	−0.021 (−0.066–0.024)	0.363			
ALB	−0.498 (−1.292–0.297)	0.218			
TBIL	−0.521 (−1.018–0.024)	**0.040**	TBIL	−0.412 (−0.854–0.030)	0.068
ALT	−0.189 (−0.454–0.077)	0.162			
CREA	0.032 (−0.140–0.205)	0.714			
URIC	0.001 (−0.029–0.031)	0.954			
LDH	−0.009 (−0.031–0.013)	0.411			
CRP	0.974 (0.391–1.557)	**0.001**	CRP	0.390 (−0.799–1.579)	0.518
PT	0.590 (−2.452–3.632)	0.702			
D-D	0.006 (0.001–0.011)	**0.011**	D-D	0.002 (−0.003–0.006)	0.465
FIB	0.942 (−1.303–3.187)	0.408			
Folate	−0.752 (−1.272–0.233)	**0.005**	Folate	−0.519 (−0.979–0.060)	**0.027**
VitB12	0.016 (0.002–0.030)	**0.030**	VitB12	0.013 (0.001–0.026)	**0.037**
GPS = 0.0	Reference				
GPS = 1.0	5.171 (−1.624–11.966)	0.135			
GPS = 2.0	16.414 (−3.657–36.484)	0.108			
mGPS = 0.0	Reference				
mGPS = 1.0	20.153 (4.789–35.518)	**0.011**	mGPS = 1.0	10.879 (−12.999–34.756)	0.369
mGPS = 2.0	20.553 (−3.489–44.596)	**0.093**	mGPS = 2.0	−6.698 (−43.607–30.211)	0.720
PNI	−0.668 (−1.316–0.020)	**0.043**	PNI	0.608 (−0.162–1.377)	0.121
NLR	3.045 (−0.029–6.120)	**0.052**	NLR	1.225 (−2.103–4.554)	0.468
PLR	0.021 (−0.041–0.083)	0.495			
LMR	−1.212 (−3.117–0.693)	0.211			
SII	0.008 (−0.004–0.019)	0.182			
UA/Cr	−0.363 (−2.303–1.577)	0.712			

*UPDRS-III, the Unified Parkinson's Disease Rating Scale Part III; PD, Parkinson's disease; HR, hazard ratio; CI, confidence interval; WBC, white blood cell; HGB, hemoglobin; PLT, platelet; ALB, albumin; TBIL, total bilirubin; ALT, alanine transaminase; Cr, creatinine; UA, uric acid; LDH, lactate dehydrogenase; CRP, C-reactive protein; PT, prothrombin time; VitB12, vitamin B12; GPS, Glasgow Prognostic Score; mGPS, modified Glasgow Prognostic Score; PNI, prognostic nutritional index; NLR, neutrophil-to-lymphocyte ratio; PLR, platelet-to-lymphocyte ratio; LMR, lymphocyte-to-monocyte ratio; SII, systemic immune-inflammation index*.

The univariate analysis identified that age, PD duration, B blood type, lymphocyte, TBIL, CRP, folate, VitB12 levels, PNI, NLR, and LMR were significant factors associated with H–Y classification. After the multivariate logistic regression analyses, we found that only age (HR = 0.049; 95% CI = 0.018–0.080; *P* = 0.002), PD duration (HR = 0.019; 95% CI = 0.013–0.026; *P* < 0.001), TBIL (HR = −0.065; 95% CI = −0.123–0.007; *P* = 0.029), folate level (HR = −0.055; 95% CI = −0.113–0.004; *P* = 0.066), and VitB12 level (HR = 0.002; 95% CI = 0.000–0.004; *P* = 0.017) were significant factors for H–Y classification (Table 5).

**Table 5:** Univariate and multivariate regression analyses of the clinical characteristics for Hoehn–Yahr (H-Y) classification of PD patients (*n* = 148).

**Univariate**	**HR (95% CI)**	***P***	**Multivariate**	**HR (95% CI)**	***P***
Gender	−0.159 (−0.752–0.433)	0.599			
Age	0.051 (0.024–0.079)	**<0.001**	Age	0.049 (0.018–0.080)	**0.002**
Education: illiteracy	−0.566 (−3.416–2.283)	0.697			
Education: primary	−1.226 (−3.793–1.342)	0.349			
Education: junior	−1.344 (−3.875–1.187)	0.298			
Education: senior	−0.852 (−3.401–1.697)	0.513			
Education: undergraduate	−1.473 (−4.012–1.067)	0.256			
Education status: post-graduate	Reference				
Smoking	−0.330 (−1.479–0.819)	0.573			
Alcohol consumption	−0.719 (−2.773–1.336)	0.493			
Diabetes mellitus	0.682 (−0.245–1.610)	0.149			
PD duration	0.022 (0.015–0.028)	**<0.001**	PD duration	0.019 (0.013–0.026)	**<0.001**
ABO blood type: A	−0.062 (−0.823–0.699)	0.873			
ABO blood type: B	0.455 (−0.271–1.181)	0.219			
ABO blood type: AB	−0.084 (−1.033–0.865)	0.862			
ABO blood type: O	Reference				
WBC	−0.008 (−0.195–0.178)	0.931			
HGB	−0.015 (−0.033–0.004)	0.124			
Lymphocyte	−0.668 (−1.267–0.069)	**0.029**	Lymphocyte	−0.347 (−1.337–0.642)	0.492
Neutrophil	0.092 (−0.139–0.322)	0.436			
Monocyte	0.870 (−0.821–2.562)	0.313			
Platelet	−0.002 (−0.006–0.003)	0.436			
ALB	−0.065 (−0.146–0.016)	0.116			
TBIL	−0.070 (−0.122–0.018)	**0.008**	TBIL	−0.065 (−0.123–0.007)	**0.029**
ALT	−0.020 (−0.047–0.007)	0.145			
CREA	−0.006 (−0.023–0.012)	0.527			
URIC	−0.001 (−0.004–0.002)	0.485			
LDH	−0.001 (−0.004–0.001)	0.224			
CRP	0.088 (0.025–0.151)	**0.006**	CRP	0.039 (−0.030–0.108)	0.269
PT	0.143 (−0.164–0.450)	0.361			
D-D	0.000 (0.000–0.001)	0.188			
FIB	0.048 (−0.178–0.274)	0.679			
Folate	−0.067 (−0.122–0.012)	**0.016**	Folate	−0.055 (−0.113–0.004)	**0.066**
VitB12	0.002 (0.000–0.003)	**0.011**	VitB12	0.002 (0.000–0.004)	**0.017**
GPS = 0.0	−1.180 (−3.227–0.866)	0.258			
GPS = 1.0	−0.505 (−2.608–1.597)	0.638			
GPS = 2.0	Reference				
mGPS = 0.0	−1.553 (−4.059–0.952)	0.224			
mGPS = 1.0	0.510 (−2.429–3.449)	0.734			
mGPS = 2.0	Reference				
PNI	−0.092 (−0.160–0.025)	**0.007**	PNI	0.035 (−0.063–0.133)	0.485
NLR	0.369 (0.051–0.687)	**0.023**	NLR	0.147 (−0.314–0.608)	0.532
PLR	0.003 (−0.003–0.010)	0.303			
LMR	−0.222 (−0.418–0.026)	**0.027**	LMR	−0.142 (−3.389–0.105)	0.259
SII	0.001 (0.000–0.002)	0.134			
UA/Cr	−0.034 (−0.229–0.162)	0.736			

*H-Y classification, Hoehn–Yahr classification; PD, Parkinson's disease; HR, hazard ratio; CI, confidence interval; WBC, white blood cell; HGB, hemoglobin; PLT, platelet; ALB, albumin; TBIL, total bilirubin; ALT, alanine transaminase; Cr, creatinine; UA, uric acid; LDH, lactate dehydrogenase; CRP, C-reactive protein; PT, prothrombin time; VitB12, vitamin B12; GPS, Glasgow Prognostic Score; mGPS, modified Glasgow Prognostic Score; PNI, prognostic nutritional index; NLR, neutrophil-to-lymphocyte ratio; PLR, platelet-to-lymphocyte ratio; LMR, lymphocyte-to-monocyte ratio; SII, systemic immune-inflammation index*.

### Changes of Outcomes According to Tertiles of Folate and VitB12

Although only a small portion of patients showed folate or VitB12 deficiency, analysis of outcomes according to folate level tertiles showed that the elevated folate level did correlate with greater decline in UPDRS-III scores (*U* = 902.500; *P* = 0.023) and H–Y classification (*U* = 781.000; *P* = 0.003) and a dramatic increase in ADL scores (*U* = 929.000; *P* = 0.048) (Table 6). However, these changes were not observed in VitB12 level tertiles related to the outcomes (Table 7).

**Table 6:** Changes of outcomes according to tertiles of Folate in PD patients (*n* = 148).

	**Folate tertile**				
**Change outcomes**	**First (<8.90 ng/ml)**	**Second (8.90–13.61 ng/ml)**	**Third (>13.61 ng/ml)**	***U***	***P***
ADL	84.90 ± 20.27	87.30 ± 19.15	91.28 ± 17.14	902.500	**0.023**
UPDRS-III	46.86 ± 16.65	43.56 ± 17.75	36.78 ± 16.91	781.000	**0.003**
H-Y classification	4.14 ± 1.98	4.06 ± 1.62	3.37 ± 1.72	929.000	**0.048**

*PD, Parkinson's disease; ADL, activity of daily living scale; UPDRS-III, the Unified Parkinson's Disease Rating Scale Part III; H-Y classification, Hoehn–Yahr classification*.

**Table 7:** Changes of outcomes according to tertiles of VitB12 in PD patients (*n* = 148).

	**VitB12 tertile**				
**Change outcomes**	**First (<236.33 pmol/L)**	**Second (236.33–339.33 pmol/L)**	**Third (>339.33 pmol/L)**	***U***	***P***
ADL	90.87 ± 14.17	88.50 ± 18.22	84.08 ± 23.15	1068.000	0.316
UPDRS-III	41.80 ± 17.35	41.10 ± 17.37	44.35 ± 17.98	1087.500	0.422
H-Y classification	3.57 ± 1.61	3.82 ± 1.81	4.18 ± 1.94	978.500	0.102

*VitB12, vitamin B12; PD, Parkinson's disease; ADL, activity of daily living scale; UPDRS-III, the Unified Parkinson's Disease Rating Scale Part III; H-Y classification, Hoehn–Yahr classification*.

### Correlation Between the Significant Factors and the Outcomes

**Figure 1:**
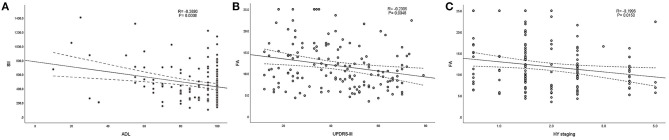
Correlation analysis between the significant factors and the outcomes. **(A)** SII was negatively associated (*P* < 0.001) with ADL score. **(B,C)** Folate level was also negatively associated with UPDRS-III score (*P* < 0.001) and H–Y classification (*P* < 0.001).

**Figure 2:**
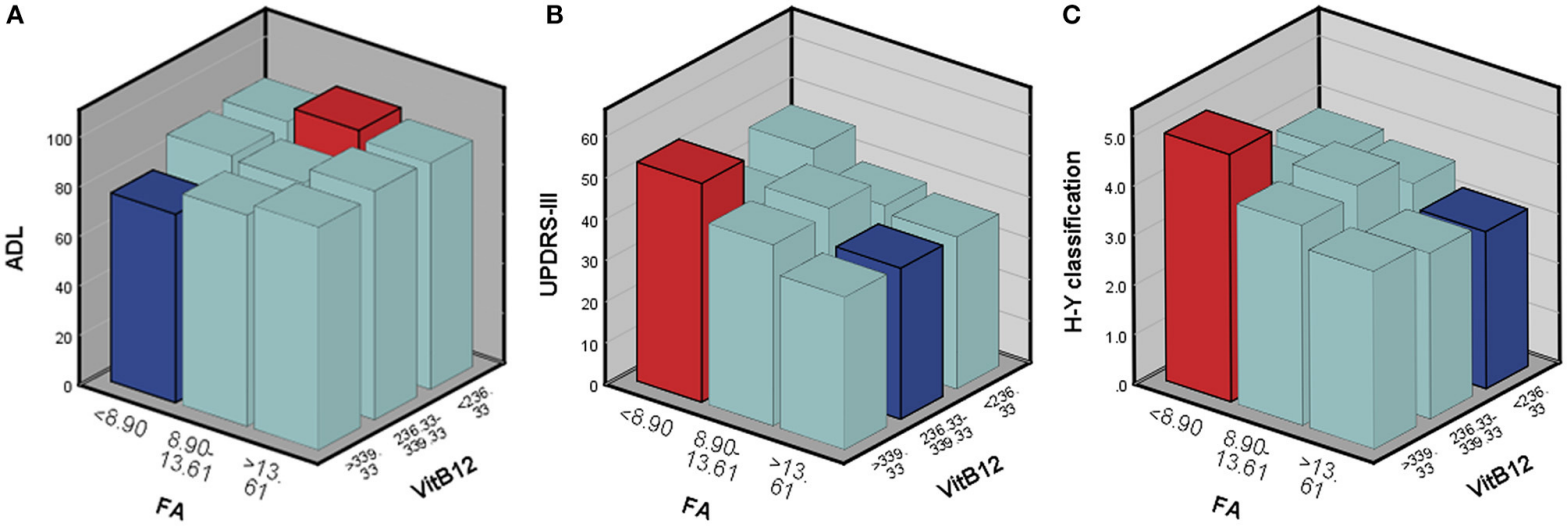
The combined effect of serum folate and VitB12 levels on the outcomes. Red bar represents the highest combined effects to the outcomes, while the blue bar represents the lowest effects. **(A)** ADL score, **(B)** UPDRS-III score, and **(C)** H-Y classification.

According to the above regression analysis and stratified analysis, we performed correlation analysis of the significant factors and the outcomes. Figure 1 shows that SII was negatively associated (*P* < 0.001) with the ADL score. Folate level was also negatively associated with UPDRS-III score (*P* = 0.005) and H–Y classification (*P* = 0.015). The three-dimensional drawing (Figure 2) showed the combined effect of folate and VitB12 levels on the outcomes. It showed that the lowest level of folate was associated with the lowest ADL score, while the lowest level of folate was related to the highest UPDRS-III score and H–Y classification. The trend between each tertile of folate levels was more stable and obvious than that of the VitB12 levels.

## Discussion

The relationship between several important inflammation-based scores and the severity of PD was first investigated in our present study. We found that PD duration and motor performance status (ADL scores and UPDRS-III scores) were highly correlated with H–Y classification stages. Folate deficiency (serum level < 4.0 ng/mL) and VitB12 deficiency (serum level < 133 pg/mL) were only present in 0.7 and 5.4% of the patients, respectively. NLR and SII were significant factors for ADL scores. Serum folate and VitB12 levels are significant factors influencing the motor performance status (ADL scores, UPDRS-III scores, and H-Y classification in PD). Dose–response analysis suggested that elevated folate level correlates with mild motor impairment in PD.

Although the underlying mechanism of neurodegeneration mediated by neuroinflammation is still rarely understood, increasing evidence suggests that the blood–brain barrier is affected under pathological conditions. Faucheux et al. (7) reported an increase in endothelial cell density in the SN of PD. Farkas et al. (8) reported pathological changes in the microanatomy of cerebral capillaries in patients with PD. Activated microglia release cytokines that may stimulate angiogenesis. Whether these cytokines can also induce brain capillary damage and changes in permeability, allowing peripheral cells, such as lymphocytes, to cross the blood–brain barrier into the brain, deserves further investigation. Studies related to the systemic inflammatory indexes have been published based on the neuroinflammation hypothesis of PD progression. Altered differential leukocyte count was involved in PD-related symptoms even though there was no sign of clinical inflammation. Relatively mild peripheral inflammation might play an important role in promoting mild disease phenotype in PD (24). A positive association between NLR and motor severity was observed in a tremor-dominant (TD) subgroup. Besides, NLR could negatively predict the striatal binding ratios in ipsilateral and contralateral putamen and caudate nuclei in a TD phenotype (25). However, other studies showed that there was no statistically significant difference between the NLR of patients with idiopathic PD (akinetic-rigid or TD subtypes) and controls (26). The present study also found that NLR was independently associated with ADL score. However, no significant association was found with UPDRS-III score and H–Y classification after multivariate analyses. As mentioned before, the complex indexes, such as PNI and SII, have proved to be more sensitive and reliable than single parameters in reflecting the immune-inflammation or nutritional status in different types of cancer (9–11) or inflammatory diseases (12). Nevertheless, these complex indexes have never been investigated in PD before. The present study showed that SII is a significant factor for ADL score. SII was negatively associated with the ADL score, indicating that patients with PD with a high SII score have a more severe motor impairment and poor performance status. The SII score, based on lymphocytes, neutrophils, and platelets, might serve as a useful tool for predicting the severity of motor impairment or prognosis in patients with PD.

The association of serum folate and VitB12 levels with PD have long been investigated, but the result regarding VitB12 level and PD was still inconsistent. Several meta-analyses have been released to address this question. Patients with PD with cognitive impairment were likely to have higher tHcy levels, lower folate, and VitB12 levels (27). However, all of the 27 included studies were conducted in China. The I-squared was extremely high across the studies, indicating heterogeneity, and the pooled result was not robust and convincible. Another meta-analysis, including 15 studies worldwide, drew a subdivided conclusion that serum tHcy level was higher in patients with PD compared with the control group, and lower folate and VitB12 levels were only observed in the PD cognitive impairment group compared with the cognitively normal group (28). On the contrary, a large community-based study found that hyperhomocysteinemia was not a risk factor for PD, as well as VitB12 and folate deficiency (29). Using CSF specimens, Christine et al. (30) found that low CSF VitB12 predicted greater worsening of the UPDRS “walking” item, whereas CSF tHcy was not associated with the progression of cognitive impairment. These findings were partially contradicted to their findings in serum, which show that low serum VitB12 at baseline predicted greater worsening of mobility, whereas elevated tHcy predicted greater cognitive decline (19). These ambiguous results were challenged by Cardoso (32). First, the metabolism of VitB12 could be induced by L-dopa (12, 13). Second, many factors might contribute to low VitB12 level. Genuine deficiency is only observed in few cases with extremely low levels. Finally, the measurement of VitB12 level should be adjusted for important confounding factors, especially the tHcy level (31, 32).

Animal model experiments showed that supplementation of folic acid could attenuate the severity of parkinsonism. While the supplement of vitamin B complex also had a beneficial effect and improved motor performance (33). Dietary folic acid provided protection against disparities in parkin mutation-induced PD *via* alleviating mitochondrial dysfunction (34). If we take up the point of view that high serum tHcy level and low serum VitB12 and folate levels were associated with progression of motor or cognitive impairment, what could be the basic underlying mechanism?—Schaffner et al. (35) uncovered that 5'-deoxyadenosylcobalamin (AdoCbl), the physiological form of VitB12, modulated PD leucine-rich repeat kinase-2 (LRRK2) kinase activity through allosteric regulation. Treatment with AdoCbl inhibited LRRK2 kinase activity and prevented neurotoxicity. AdoCbl also alleviated deficits in dopamine release sustainability caused by LRRK2 disease variants in mouse models (35).

Based on the ambiguous epidemiological studies and positive fundamental research, the plan for supplementation of folic acid or VitB12 might come up and be put into practice. However, no significant association was observed for dietary intake of folic acid and VitB12 with the risk of developing PD (36). Another large longitudinal study on patients with early PD showed that no significant differences in the progress of UPDRS scores were observed. However, a well-designed prospective nested case–control study showed that a low–folate density diet, 2–8 years before PD diagnosis, was significantly associated with olfactory dysfunction at the time of PD diagnosis (37).

The unbalanced gender distribution was observed in demographics, which was contradictory to the previous study (38). PD is usually more common in men than women, with a male-to-female ratio of about 1.5. The underlying mechanism is rarely understood. Possible explanations for this difference include more frequent occupational exposures in men, neuroprotection from estrogens in women, and X-linked genetic factors. However, gender is neither considered a protective factor nor a risk factor in an epidemiological study of PD. There might be recall and selection bias in the retrospective study. Based on the univariate or multivariate analysis in the present study, gender was not statistically significant. It is widely accepted that UA is a natural antioxidant that has protective effects of scavenging free radicals and antioxidation in neurological disorders. It has been reported that lower serum UA level was associated with the worsening of motor function (39). Serum UA and UA/Cr levels were negatively correlated with the H–Y classification of PD and are independent of negatively predicting biological indexes of PD incidence and progression (40). However, the univariate and multivariate analyses of the study did not reveal significant associations between serum UA and UA/Cr levels and the motor performance status of PD.

There are several limitations in the present study: First, this is a retrospective study design, where recall and selection bias might exist. Second, age- and gender-matched healthy controls are not included because the elderly or retired people seldom go for medical check-ups, and the conventional check-ups do not contain tests for folate or VitB12 levels and for the assessment of motor performance status or cognitive ability. Third, a powerful endpoint assessment tool, such as UPDRS-II, was not used. Fourth, adjustments for important confounding factors, such as tHcy levels, medication, and gastrectomy history, were not performed.

## Conclusions

The present study indicates that serum folate, VitB12 levels, and SII are significant factors influencing the status of motor performance in PD. SII is negatively associated with the ADL. Elevated folate level correlates with mild motor impairment in PD. Well-designed (nested case–control, propensity score matching, or long-time follow-up prospective design), large sample size studies would be needed to further clarify the influence of these factors.

## Data Availability Statement

The raw data supporting the conclusions of this article will be made available by the authors, without undue reservation.

## Ethics Statement

The studies involving human participants were reviewed and approved by the Ethics Committee of the Third Affiliated Hospital of Guangzhou Medical University and the Ethics Committee of Guangdong Provincial People's Hospital (No. 2021-006). The patients/participants provided their written informed consent to participate in this study.

## Author Contributions

SL and LW designed the whole study and revised the manuscript. SL, QZ, and YL enrolled patients and extracted raw data. KN and YZ performed the statistical analyses and prepared tables and figures. SL wrote the manuscript. All authors read and approved the final manuscript.

## Conflict of Interest

The authors declare that the research was conducted in the absence of any commercial or financial relationships that could be construed as a potential conflict of interest.

## References

[1] Rossi A Berger K Chen H Leslie D Mailman RB Huang X. Projection of the prevalence of Parkinson's disease in the coming decades: revisited. Mov Disord. (2018) 33:156–9. 10.1002/mds.27063 28590580 PMC5720940

[2] Moore DJ West AB Dawson VL Dawson TM. Molecular pathophysiology of Parkinson's disease. Annu Rev Neurosci. (2005) 28:57–87. 10.1146/annurev.neuro.28.061604.135718 16022590

[3] Michel PP Hirsch EC Hunot S. Understanding dopaminergic cell death pathways in parkinson disease. Neuron. (2016) 90:675–91. 10.1016/j.neuron.2016.03.038 27196972

[4] Przedborski S. The two-century journey of Parkinson disease research. Nat Rev Neurosci. (2017) 18:251–9. 10.1038/nrn.2017.25 28303016

[5] Hirsch EC Hunot S. Neuroinflammation in Parkinson's disease: a target for neuroprotection? Lancet Neurol. (2009) 8:382–97. 10.1016/S1474-4422(09)70062-6 19296921

[6] Li S Nie K Zhang Q Guo M Qiu Y Li Y. Macrophage migration inhibitory factor mediates neuroprotective effects by regulating inflammation, apoptosis and autophagy in parkinson's disease. Neuroscience. (2019) 416:50–62. 10.1016/j.neuroscience.2019.05.052 31170483

[7] Faucheux BA Bonnet AM Agid Y Hirsch EC. Blood vessels change in the mesencephalon of patients with Parkinson's disease. Lancet. (1999) 353:981–2. 10.1016/S0140-6736(99)00641-8 10459912

[8] Farkas E De Jong GI de Vos RA Jansen SE Luiten PG. Pathological features of cerebral cortical capillaries are doubled in Alzheimer's disease and Parkinson's disease. Acta Neuropathol. (2000) 100:395–402. 10.1007/s004010000195 10985698

[9] Hu B Yang XR Xu Y Sun YF Sun C Guo W. Systemic immune-inflammation index predicts prognosis of patients after curative resection for hepatocellular carcinoma. Clin Cancer Res. (2014) 20:6212–22. 10.1158/1078-0432.CCR-14-0442 25271081

[10] Huang H Liu Q Zhu L Zhang Y Lu X Wu Y. Prognostic value of preoperative systemic immune-inflammation index in patients with cervical cancer. Sci Rep. (2019) 9:3284. 10.1038/s41598-019-39150-0 30824727 PMC6397230

[11] Jan HC Yang WH Ou CH. Combination of the preoperative systemic immune-inflammation index and monocyte-lymphocyte ratio as a novel prognostic factor in patients with upper-tract urothelial carcinoma. Ann Surg Oncol. (2019) 26:669–84. 10.1245/s10434-018-6942-3 30374917

[12] Erdogan T. Role of systemic immune-inflammation index in asthma and NSAID-exacerbated respiratory disease. Clin Respir J. (2020) 15:400–5. 10.1111/crj.13314 33249745

[13] Green R Allen LH Bjørke-Monsen AL Brito A Guéant JL Miller JW. Vitamin B(12) deficiency. Nat Rev Dis Primers. (2017) 3:17040. 10.1038/nrdp.2017.40 28660890

[14] Kruman II Kumaravel TS Lohani A Pedersen WA Cutler RG Kruman Y. Folic acid deficiency and homocysteine impair DNA repair in hippocampal neurons and sensitize them to amyloid toxicity in experimental models of Alzheimer's disease. J Neurosci. (2002) 22:1752–62. 10.1523/JNEUROSCI.22-05-01752.2002 11880504 PMC6758871

[15] Smith AD Refsum H. Homocysteine, B vitamins, and cognitive impairment. Annu Rev Nutr. (2016) 36:211–39. 10.1146/annurev-nutr-071715-050947 27431367

[16] Lee CC Hsu SW Huang CW Chang WN Chen SF Wu MK. Effects of Homocysteine on white matter diffusion parameters in Alzheimer's disease. BMC Neurol. (2017) 17:192. 10.1186/s12883-017-0970-7 28985720 PMC5639619

[17] de Lau LM Smith AD Refsum H Johnston C Breteler MM. Plasma vitamin B12 status and cerebral white-matter lesions. J Neurol Neurosurg Psychiatry. (2009) 80:149–57. 10.1136/jnnp.2008.149286 18977824

[18] de Lau LM Koudstaal PJ Witteman JC Hofman A Breteler MM. Dietary folate, vitamin B12, and vitamin B6 and the risk of Parkinson disease. Neurology. (2006) 67:315–8. 10.1212/01.wnl.0000225050.57553.6d 16864826

[19] Christine CW Auinger P Joslin A Yelpaala Y Green R. Vitamin B12 and homocysteine levels predict different outcomes in early parkinson's disease. Mov Disord. (2018) 33:762–70. 10.1002/mds.27301 29508904

[20] Scalabrino G Veber D Mutti E. Experimental and clinical evidence of the role of cytokines and growth factors in the pathogenesis of acquired cobalamin-deficient leukoneuropathy. Brain Res Rev. (2008) 59:42–54. 10.1016/j.brainresrev.2008.05.001 18538413

[21] Scalabrino G Peracchi M. New insights into the pathophysiology of cobalamin deficiency. Trends Mol Med. (2006) 12:247–54. 10.1016/j.molmed.2006.04.008 16690356

[22] Yi Y Ding L Wen H Wu J Makimoto K Liao X. Is barthel index suitable for assessing activities of daily living in patients with dementia? Front Psychiatry. (2020) 11:282. 10.3389/fpsyt.2020.00282 32457659 PMC7225343

[23] Saranza G Lang AE. Levodopa challenge test: indications, protocol, and guide. J Neurol. (2020). 10.1007/s00415-020-09810-7. [Epub ahead of print]. 32333167

[24] Umehara T Oka H Nakahara A Matsuno H Murakami H. Differential leukocyte count is associated with clinical phenotype in Parkinson's disease. J Neurol Sci. (2020) 409:116638. 10.1016/j.jns.2019.116638 31865186

[25] Sanjari MH Ghazi SF Mojtahed ZM Ashraf-Ganjouei A Aarabi MH. Association between peripheral inflammation and DATSCAN data of the striatal nuclei in different motor subtypes of parkinson disease. Front Neurol. (2018) 9:234. 10.3389/fneur.2018.00234 29713303 PMC5911462

[26] Ataç UC Gökçe ÇB Ünal AH Inan LE Yoldaş TK. Comparison of neutrophil-lymphocyte ratio (NLR) in Parkinson's disease subtypes. Neurol Sci. (2017) 38:287–93. 10.1007/s10072-016-2758-8 27837368

[27] Xie Y Feng H Peng S Xiao J Zhang J. Association of plasma homocysteine, vitamin B12 and folate levels with cognitive function in Parkinson's disease: a meta-analysis. Neurosci Lett. (2017) 636:190–5. 10.1016/j.neulet.2016.11.007 27840145

[28] Dong B Wu R. Plasma homocysteine, folate and vitamin B12 levels in Parkinson's disease in China: a meta-analysis. Clin Neurol Neurosurg. (2020) 188:105587. 10.1016/j.clineuro.2019.105587 31733593

[29] Wei Z Tiandong W Yang L Huaxing M Guowen M Yalan F. Parkinson's disease and homocysteine: a community-based study in a folate and vitamin B12 deficient population. Parkinsons Dis. (2016) 2016:9539836. 10.1155/2016/9539836 27656311 PMC5021874

[30] Christine CW Auinger P Saleh N Tian M Bottiglieri T Arning E. Relationship of cerebrospinal fluid vitamin B12 status markers with Parkinson's disease progression. Mov Disord. (2020) 35:1466–71. 10.1002/mds.28073 32407590 PMC7496300

[31] Stabler SP. Vitamin B12 deficiency. N Engl J Med. (2013) 368:2041–2. 10.1056/NEJMc1304350 23697526

[32] Cardoso F. Vitamin B12 and Parkinson's disease: what is the relationship? Mov Disord. (2018) 33:702–3. 10.1002/mds.27366 29508925

[33] Haghdoost-Yazdi H Fraidouni N Faraji A Jahanihashemi H Sarookhani M. High intake of folic acid or complex of B vitamins provides anti-Parkinsonism effect: no role for serum level of homocysteine. Behav Brain Res. (2012) 233:375–81. 10.1016/j.bbr.2012.05.011 22610053

[34] Srivastav S Singh SK Yadav AK Srikrishna S. Folic acid supplementation ameliorates oxidative stress, metabolic functions and developmental anomalies in a novel fly model of Parkinson's disease. Neurochem Res. (2015) 40:1350–9. 10.1007/s11064-015-1598-x 25963948

[35] Schaffner A Li X Gomez-Llorente Y Leandrou E Memou A Clemente N. Vitamin B(12) modulates Parkinson's disease LRRK2 kinase activity through allosteric regulation and confers neuroprotection. Cell Res. (2019) 29:313–29. 10.1038/s41422-019-0153-8 30858560 PMC6462009

[36] Shen L. Associations between B vitamins and Parkinson's disease. Nutrients. (2015) 7:7197–208. 10.3390/nu7095333 26343714 PMC4586528

[37] Håglin L Johansson I Forsgren L Bäckman L. Intake of vitamin B before onset of Parkinson's disease and atypical parkinsonism and olfactory function at the time of diagnosis. Eur J Clin Nutr. (2017) 71:97–102. 10.1038/ejcn.2016.181 27703161

[38] Taylor KS Cook JA Counsell CE. Heterogeneity in male to female risk for Parkinson's disease. J Neurol Neurosurg Psychiatry. (2007) 78:905–6. 10.1136/jnnp.2006.104695 17635983 PMC2117744

[39] Sleeman I Lawson RA Yarnall AJ Duncan GW Johnston F Khoo TK. Urate and homocysteine: predicting motor and cognitive changes in newly diagnosed Parkinson's disease. J Parkinsons Dis. (2019) 9:351–9. 10.3233/JPD-181535 30909247 PMC6597987

[40] Zhong LL Song YQ Tian XY Cao H Ju KJ. Level of uric acid and uric acid/creatinine ratios in correlation with stage of Parkinson disease. Medicine. (2018) 97:e10967. 10.1097/MD.0000000000010967 29952939 PMC6039589

